# Development of a World Health Organization indicator and corresponding questions to measure effective coverage of rehabilitation

**DOI:** 10.1016/j.eclinm.2025.103317

**Published:** 2025-07-01

**Authors:** Wouter De Groote, Pierre Côté, Jessica J. Wong, Sheilah Hogg-Johnson, Tara Chen, Linamara Battistella, George K.C. Wong, Jan Hartvigsen, Jill A. Hayden, Rajani Mullerpatan, Farooq A. Rathore, Hana Alsobayel, Margareta Nordin, Dan Kajungu, Jae-Young Lim, Rafael Lozano, Antony Duttine, Melanie Cowan, Leanne Riley, Somnath Chatterji, Alarcos Cieza

**Affiliations:** a Sensory Functions, Disability and Rehabilitation Unit, Department for Noncommunicable Diseases, Rehabilitation and Disability, World Health Organization, Avenue Appia 20, 1211, Geneva, Switzerland; b Institute for Disability and Rehabilitation Research, Faculty of Health Sciences, Ontario Tech University, 2000 Simcoe Street North, Oshawa, Ontario, Canada; c Department of Research and Innovation, Canadian Memorial Chiropractic College, 6100 Leslie Street, Toronto, Ontario, Canada; d Department of Rehabilitation Medicine, The Seventh Affiliated Hospital, Sun Yat-Sen University, China; e Department of Physical Medicine and Rehabilitation, Faculty of Medicine, University of São Paulo, Brazil; f The Hong Kong Society for Rehabilitation, Jockey Club Geriatric Rejuvenation Hub, 7 Sha Wan Drive, Pokfulam, Hong Kong; g Center for Muscle and Joint Health, Department of Sports Science and Clinical Biomechanics, University of Southern Denmark, Campusvej 55, 5230, Odense M, Denmark; h Chiropractic Knowledge Hub, Camusvej 55, 5230, Odense M, Denmark; i Department of Community Health & Epidemiology, Dalhousie University, 5790 University Avenue, B3H 1V7, Halifax, Nova Scotia, Canada; j School of Physiotherapy, Center of Human Movement Science, Mahatma Gandhi Mission Institute of Health Sciences, Navi Mumbai, India; k Armed Forces Institute of Rehabilitation Medicine, Abid Majeed Road, Rawalpindi, 46000, Pakistan; l Department of Rehabilitation Sciences, College of Applied Medical Sciences, King Saud University, Riyadh, Saudi Arabia; m Departments of Orthopedic Surgery and Environmental Medicine, New York University, New York, NY, USA; n Makerere University Centre for Health and Population Research & Iganga Mayuge HDSS, Makerere University, P.O Box 111, Iganga, Kampala, Uganda; o Department of Global Health, Stellenbosch University, Stellenbosch, South Africa; p Department of Rehabilitation Medicine, Seoul National University College of Medicine, Seoul National University Bundang Hospital, 82 Gumiro 173, Bundang-gu, Seongnam, South Korea; q Professor Emeritus, Health Metrics Sciences, Institute for Health Metrics and Evaluation, School of Medicine, University of Washington, 3980 15th Ave. NE, Seattle, WA, 98195, USA; r School of Medicine, National Autonomous University of Mexico, Mexico City, 04510, Mexico; s Surveillance, Monitoring and Reporting Unit, Department for Noncommunicable Diseases, Rehabilitation and Disability, World Health Organization, Avenue Appia 20, 1211, Geneva, Switzerland; t Former DNA Unit Head, Department for Data, Analytics, and Delivery for Impact, World Health Organization, Avenue Appia 20, 1211, Geneva, Switzerland

**Keywords:** Rehabilitation, Effective coverage, Universal health coverage, Health system performance, Health services, Low back pain

## Abstract

**Background:**

Rehabilitation services are essential health services that should be made available to a population. Measuring effective coverage requires the assessment of whether a population's health services needs are met and whether they are met through quality interventions that produce the desired health gain. We propose a global indicator and corresponding questions to measure effective coverage of rehabilitation through population-based surveys.

**Methods:**

An indicator to measure effective coverage of rehabilitation requires a clear definition of rehabilitation service need, utilization, and quality. These terms are defined for a tracer health condition with impact on functioning and for which rehabilitation services are beneficial. We selected chronic primary low back pain as the tracer health condition. Following technical input from experts early 2023, we drafted and cognitively tested a set of questions from August till November 2023 to provide key data points for calculating the number of people living with chronic primary low back pain who received rehabilitation services. To determine whether quality rehabilitation services have been delivered, the health gain or benefit can be measured using a valid functioning measure with a known Minimal Important Change value, i.e. a minimal improvement that is meaningful to the client. We selected the shorter version of World Health Organization Disability Assessment Schedule 2.0 to meet this criterion.

**Findings:**

The proposed global indicator is defined as the proportion of adults with chronic primary LBP experiencing limitations in functioning that benefited from rehabilitation. There are eight corresponding questions to calculate the number of adults with chronic primary LBP experiencing limitations in functioning and utilizing rehabilitation services. The assessment of a benefit of received rehabilitation services is based on a change in functioning that is greater than the Minimal Important Change measured with World Health Organization Disability Assessment Schedule 2.0 12-item (simple scoring). The Minimal Important Change was set at 6 points following a secondary analysis of studies reporting on rehabilitation outcomes for people with chronic low back pain.

**Interpretation:**

We propose a global tracer indicator for measuring effective coverage of rehabilitation at the population level that is captured through population-based surveys. This global indicator uses chronic primary low back pain as the tracer health condition and World Health Organization Disability Assessment Schedule 2.0 12-item to assess whether quality interventions have been provided that produce the desired health gain.

**Funding:**

This research received no specific grant from any funding agency in the public, commercial or not-for-profit sectors.

**Research in context**

**Evidence before this study**

The WHO universal health coverage (UHC) measurement framework, and other frameworks that have followed, does not include a rehabilitation indicator yet. Also, the mapping of country effective coverage indicators to a matrix representing health service types by the Global Burden of Disease 2019 Universal Health Coverage Collaborators did not include rehabilitation.

**Added value of this study**

This paper presents the first global tracer indicator for effective coverage of rehabilitation at population level to be captured through population-based surveys with corresponding questions. It uses chronic primary low back pain as the tracer health condition and the World Health Organization Disability Assessment Schedule 2.0 12-item to assess whether quality interventions have been provided that resulted in the intended health benefit.

**Implications of all the available evidence**

Further field testing for the indicator and evaluation of corresponding questions are recommended. When reliable data become available, rehabilitation coverage could be included in any measurement framework assessing country progress towards UHC. In addition, this development could be studied to expand to other rehabilitation interventions or health conditions that require rehabilitation and thereby make the assessment of the health system for rehabilitation more comprehensive.

## Introduction

In February 2017, the World Health Organization (WHO) launched the Rehabilitation 2030 Call for Action which draws attention to the profound unmet need for rehabilitation worldwide and to the importance of strengthening health systems to provide rehabilitation. Rehabilitation services are essential health services to optimize functioning and reduce disability in individuals with health conditions in interaction with their environment^1^; hence, rehabilitation is a critical health strategy to improve individual and population health and well-being, adding to societal welfare.^2^ Many people, however, have limited access to the required rehabilitation services, particularly in low- and middle-income countries (LMIC).^3^

Globally, in 2021, 2.6 billion individuals were living with a health condition that could benefit from rehabilitation, contributing to 340 million Years Lived with Disability (YLD). This number has increased by 79.4% from 1990 to 2021.^4^ People with musculoskeletal, neurologic, respiratory, cardiovascular, mental and other health conditions can all be potential beneficiaries from rehabilitation. Rehabilitation services are provided by a team of professionals such as occupational therapists, physiotherapists, orthotics and prosthetics, speech and language therapists, chiropractors, rehabilitation physicians and nurses, psychologists, and others. Given the large number of health conditions that are amenable to rehabilitation, it is important to recognize that several rehabilitation models of care exist. To serve all in need, the strengthening of all levels of the health system is required.^5^

At the 76th World Health Assembly in May 2023, WHO Member States endorsed the Resolution “Strengthening rehabilitation in health systems”. The resolution urges countries “to expand rehabilitation to all levels of health, from primary to tertiary care, and to ensure the availability and affordability of quality and timely rehabilitation services”.^6^ It also requests the Director-General of WHO to develop feasible global rehabilitation targets and indicators for effective coverage of rehabilitation services.

Measuring effective coverage is recommended when assessing health system performance and monitoring progress towards achieving universal health coverage (UHC).^7^, ^8^, ^9^, ^10^ The measurement of effective coverage requires that we assess whether a population's health services needs are actually met through quality interventions that produce the desired health gain.^11^, ^12^, ^13^ Therefore, effective coverage is defined as the proportion of potential health gain that is delivered to the population through the health system. This is particularly useful for decision makers because it measures a health outcome that can be related with the health budget. In contrast to crude coverage, which focuses solely on intervention access or utilization, effective coverage is a measure that unites intervention need, utilization, and quality.^14^ For example, effective coverage for malaria case management ranges from 8% to 72% in Sub-Saharan African countries due to health system factors such as treatment seeking, provider compliance, adherence, and quality of medication.^15^ Some other examples include the effective coverage measurements for cataract surgery, refractive error services, newborn hearing screening services, hearing technology use among adults with hearing loss, blood pressure control among people with hypertension and glycaemic control among people with diabetes.^16^, ^17^, ^18^ To estimate effective coverage for a specific health intervention at a population level, data from individuals are aggregated.

We propose a global indicator to measure effective coverage of rehabilitation for a tracer health condition and corresponding questions for capturing the indicator through population-based surveys.^19^ This paper describes the indicator proposal and the co-development of a set of questions that provides the data that are needed to calculate the indicator.

## Methods

### Indicator proposal

This section describes the approach for defining the three components required in the measurement of effective coverage of rehabilitation, namely, rehabilitation services need, utilization, and quality. Rehabilitation services need refers to the population in need of a particular rehabilitation service; utilization refers to the utilization of these services; and quality refers to the actual health gain resulting from receipt of services (the outcome of services).^13^ The population in need of rehabilitation services (who would benefit from receiving rehabilitation interventions) defines the denominator of the indicator. The numerator is defined by the number of people receiving rehabilitation services with sufficient quality to produce a meaningful improvement in health. The utilization of quality rehabilitation services, hence, is defined conditional on need of these services.^20^

There is a wide range of health conditions amenable to rehabilitation and therefore a long list of rehabilitation interventions exists to manage those conditions.^21^ When defining the population that would benefit from rehabilitation to constitute the denominator of our effective coverage indicator (component ‘need’), however, one cannot monitor all amenable conditions. For rehabilitation, as for many areas of health, the estimate of effective coverage must rely on selecting one or more health conditions (called tracer health conditions) that meet clear criteria. First, it is important to prioritize health needs based on the condition's impact which, for rehabilitation, may be defined as Years Lived with Disability (YLD) due to the health condition.^22^ Second, the selection of the tracer health condition should be based on the availability of effective interventions that are recommended in clinical practice guidelines.^21^ A high percentage of people with the condition should be able to benefit from rehabilitation services. Finally, because our indicator will be captured through a population survey, the tracer health condition must be clearly defined and be prevalent enough so that a reliable estimate can be produced.

Based on the above criteria, we selected chronic non-specific or primary^23^ low back pain (LBP) as the tracer health condition. Globally, the highest contribution to the need for rehabilitation is related to musculoskeletal disorders, with LBP being the most prevalent condition (568 million people) and in most countries (134 of the 204 countries analyzed), and responsible for 7.4% of global Years Lived with a Disability (YLD).^4^ Up to 95% of LBP cases are defined as primary LBP, for which a specific diagnosis cannot be made with certainty.^24^ Up to 30% of LBP cases may develop chronic LBP,^25^ defined as pain lasting 12 weeks or more.^26^^,^^27^ Chronic LBP affects about 10–19.6% of individuals aged between 20 and 59 years,^28^, ^29^, ^30^ while its prevalence in West Africa has been estimated as high as 35.5%.^31^ Chronic LBP can have an important impact on functioning and health-related quality of life^32^; in fact, it is a main cause of disability worldwide.^33^ It is associated with reduced working capacity and sick leave, high health care utilization and costs, especially when higher levels of disability are experienced.^34^ The costs associated with loss of productivity are substantial; the overall prevalence of chronic LBP in LMIC is estimated to be around 52% in workers.^35^^,^^36^

Available evidence suggests that many rehabilitation interventions are cost-effective for the management of sub-acute and chronic LBP.^37^, ^38^, ^39^, ^40^, ^41^, ^42^, ^43^, ^44^, ^45^ In particular, multidisciplinary rehabilitation that includes physical exercises combined with psychological component or social/work targeted component is effective.^46^, ^47^, ^48^ Interventions for the rehabilitation management of chronic primary LBP have recently been recommended in WHO publications.^18^^,^^49^

The monitoring of rehabilitation service use for people living with chronic primary LBP could involve questions to capture those with the condition and among these people, identify those who received (versus have not received) rehabilitation services. When measuring utilization of rehabilitation services, questions that focus on accessing services from specific rehabilitation professions may not be appropriate because of country-level variations on the availability of professions. This is particularly relevant to LMIC where task-sharing approaches may be used. Furthermore, it is unclear whether asking about ‘receiving (medical) rehabilitation services’ will be equally well understood in different countries and health systems. A way forward is to define rehabilitation services utilization as accessing any health service provider that aimed to improve a respondent's functioning. In addition, receiving key interventions in the management of the condition contribute to the definition of access to rehabilitation.

To measure effective coverage, it is necessary to determine whether the rehabilitation intervention is associated with the intended health benefit. This requirement is generally measured by a biological marker or measurable health outcome.^7^ Because rehabilitation is a “set of interventions designed to optimize functioning and reduce disability in individuals with health conditions in interaction with their environment”,^1^ it is logical to use functioning as the health outcome. Clinical goals of rehabilitation are an improvement in functioning as a measure of improvement in health and not necessarily curing the underlying health condition.^50^^,^^51^

Measuring a benefit from rehabilitation interventions based on the evaluation of functioning before and after receiving the target rehabilitation intervention compared to a control intervention is not possible in the context of a cross-sectional population survey. However, when using population survey data, the evaluation of benefits of an intervention can be done by comparing the self-reported health outcomes of individuals with and without exposure to the intervention.^7^ A health benefit from rehabilitation can then be defined as an improvement in functioning based on the Minimal Important Change (MIC), a value that represents a minimal difference or improvement that is meaningful to the patient,^52^ for a given functioning measure.

### Development of questions

Draft questions were prepared early 2023 by the WHO Rehabilitation Programme to estimate the need and utilization of services in the population assessed.

First, questions were developed to define the indicator's denominator by identifying the number of people living with the tracer health condition (‘need’ component). Health needs at the individual level, in surveys, can be measured in three ways: based on normative criteria (e.g. all children under five years of age), self-reported symptoms, or with biometric measurements. Of these three, the measurement of a health need based on self-reported symptoms has the weakest face validity but may be the preferred option for some health conditions.^53^ A standardized and internationally accepted definition of the tracer health condition is needed for this; chronic LBP is defined as pain and discomfort for more than three months located to the lumbar region or gluteal region as anatomically outlined from the 12th thoracic vertebra to the gluteal folds, with or without radiating leg pain.^54^ Pain intensity may fluctuate during this episode of pain. For our purpose, questions from the Global Alliance for Musculoskeletal Health Survey^55^ were adapted to identify respondents with LBP and to determine whether functioning difficulties have resulted from the condition. The standard recall period ‘during the last 12 months’ from the World Health Survey^56^ was included. A question to confirm the duration of the pain of more than three months was newly developed. Lastly, some remaining respondents (0.7–4.5%) would still have a specific cause for their LBP related to inflammatory disease, osteoporotic fractures, failed back surgery or malignancy.^24^ A further distinction between these specific causes of LBP and primary (or non-specific) LBP would, however, need to involve questions asking about a specific diagnosis received, with a risk of misclassification, or require a thorough clinical history record and examination. It was decided to accept this minor misclassification for chronic primary LBP but, at least, to exclude potential chronic LBP cases resulting from failed back surgery with one additional question.

Second, for the numerator, questions are needed to assess whether identified individuals with chronic primary LBP received rehabilitation (‘utilization’ component). With our definition of rehabilitation services utilization in mind, a review of existing population survey questions that determine access to rehabilitation, available from a recent systematic review, has been conducted.^57^ No survey question was identified that suited our approach for defining access to rehabilitation. Hence, we developed questions to confirm whether the individual consulted a health service provider who aimed to improve the functioning or who provided at least one key rehabilitation intervention for the management of chronic primary LBP. Key interventions include counselling about pain and physical activities, psychological and exercise recommendations.^21^ To confirm access to rehabilitation is for chronic low back pain, and not for acute pain, an additional question was developed to make the distinction: the respondent is first asked about treatment in the initial phase of the pain period, and then moves on to the questions determining access to rehabilitation for the chronic phase.

Mid 2023 the draft questions were presented for feedback to the Surveillance, Monitoring and Reporting unit of the Department for Noncommunicable Diseases, Rehabilitation and Disability, at WHO, Geneva, Switzerland, and to an external advisory group (see Contributors’ section below) of epidemiologists, rehabilitation academics and health system specialists from the WHO Rehabilitation Programme network (WHO experts from the Package of Interventions for Rehabilitation of LBP and WHO Collaborating Centers for Rehabilitation). Members of the external advisory group underwent a WHO clearance process for conflicts of interest. The process involved online meetings outlining the measurement components and corresponding questions with opportunities for providing more feedback in writing following the meetings. Suggestions for improving the face validity of the questions and response options and for improving the data collection flow were incorporated by the members of the WHO Rehabilitation Programme.

From August till November 2023, an improved version of the questions underwent cognitive testing^58^ in a sample of 29 respondents with chronic LBP that have been recruited from available patients in the following WHO Collaborating Centers for Rehabilitation; the Hong Kong Society for Rehabilitation, Sun Yat-Sen University in China (CHN-50), and the Faculty of Medicine of the University of São Paulo, Brazil. Cognitive testing included the following steps: briefing and training of a local researcher that will conduct the cognitive testing, forward and backward translation of the questions by two independent bilingual translators, recruitment of respondents among available patients including informed consent forms signed, cognitive testing using the think-aloud approach,^59^ preparing for debrief by comparing and summarizing the experiences and any concerns that arose in a brief cognitive testing report using anonymized information and according to four themes of testing (administration, interpretation, responses, and consistency), and a debriefing session with the WHO Rehabilitation Programme representative. There was no recording of the cognitive testing interview, just note-taking by the local researcher. Interviewers nor respondents were remunerated for their participation. This process allowed for confirming the face validity of the questions and response options.

To assess a benefit from rehabilitation, the shorter version of World Health Organization Disability Assessment Schedule (WHODAS) 2.0 was selected (simple scoring with a maximum of 48). It is a 12-item generic measure used to score health-related disability and functioning based on the International Classification of Functioning, Disability and Health (ICF). WHODAS 2.0 12-item can be used in clinical and population-based settings as an outcome measure in effectiveness studies.^60^ This functioning measure is easy and quick to administer and has good psychometric properties in persons with chronic LBP. Evidence suggests that the WHODAS 2.0 12-item has adequate structural validity and internal consistency in persons with LBP. The longer version of WHODAS 2.0 (36-item) has adequate content validity, test-retest reliability, and interrater reliability in persons with LBP.^61^ The evidence also suggests that the WHODAS 2.0 36-item has adequate responsiveness in persons with LBP.^62^, ^63^, ^64^ The responsiveness of the WHODAS 2.0 36-item and 12-item is further supported by other studies of samples of patients with other chronic conditions, such as rheumatoid arthritis and Parkinson's disease.^65^^,^^66^ Although no floor or ceiling effects were observed for the WHODAS 2.0 36-item in patients with chronic LBP,^67^ a potential floor effect cannot be ruled out for the WHODAS 2.0 12-item.^61^

### Patient and public involvement

Patients and/or the public were not involved in the design, or conduct, or reporting of this research.

### Ethics

This study involved people with lived experience who were consulted for input on the questions, and the WHO Ethics Committee decided that this was exempt from ethics approval. No patient data were collected. Informed consent forms were obtained from all participants.

### Role of the funding source

This research received no specific grant from any funding agency in the public, commercial or not-for-profit sectors.

## Results

**Fig. 1:**
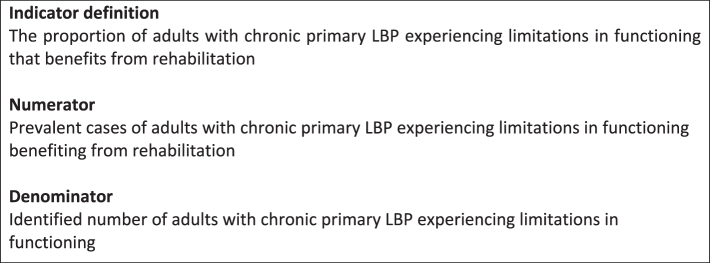
Indicator of effective coverage of rehabilitation.

The source population for our indicator includes adults with chronic primary LBP, with or without radiating leg pain, experiencing limitations in functioning. The indicator used to measure effective coverage of rehabilitation is defined as the proportion of adults with chronic primary LBP experiencing limitations in functioning that benefits from rehabilitation (Fig. 1). The indicator can be reported as two metrics: 1) Crude coverage: the percentage of adults with chronic primary LBP experiencing limitations in functioning who received rehabilitation, and 2) Effective coverage: the percentage of adults with chronic primary LBP experiencing limitations in functioning who received and benefited from rehabilitation. We recommend stratifying the indicator according to age, gender, socio-economic status, urban/rural, ethnicity, and other relevant sociodemographic factors, where available.

**Fig. 2:**
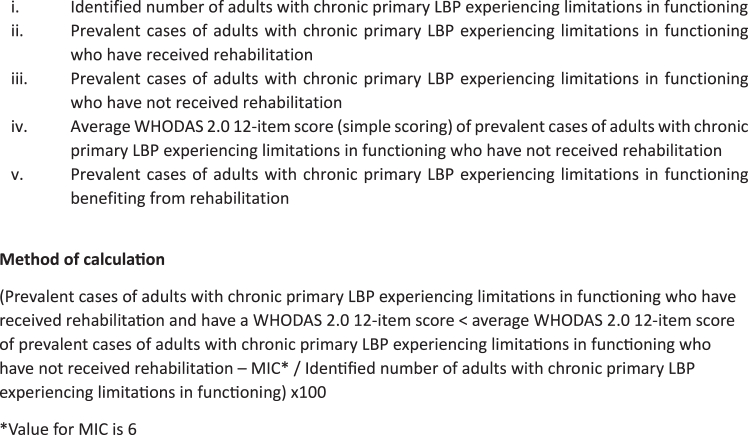
Key data points and calculation of the indicator.

The key data points required in the calculation of the indicator and the method of calculation are shown at Fig. 2. To identify respondents benefiting from rehabilitation, the average WHODAS 2.0 12-item score of cases who did not receive rehabilitation is imputed as the baseline score for those receiving rehabilitation. Respondents benefiting from rehabilitation should achieve a WHODAS 2.0 12-item score that is lower than the baseline WHODAS 2.0 12-item score minus the MIC. This is taking into account that people not accessing rehabilitation may have higher levels of functioning compared to those who are accessing rehabilitation^34^ but, on the other hand, are more likely to have a poorer self-rated health^68^ and therefore a lower level of functioning^69^ when experiencing difficulties with accessing rehabilitation.

We propose a MIC value of 6 for this indicator as measured using the WHODAS 2.0 12-item given the following considerations. A recent systematic review identified one study reporting the Minimal Important Change (MIC) for the WHODAS 2.0 12-item.^61^ In this cross-sectional study, the MIC ranged from 3.09 to 4.68/48 using a distribution-based method.^70^ However, according to the COnsensus-based Standards for the selection of health Measurement INstruments (COSMIN), a preferred method to compute MICs is the anchor-based approach which integrates patients’ consideration of important differences.^71^ Using the Physical Functioning domain of the Short Form-36 and Oswestry Disability Index (ODI) as anchors, a secondary analysis of previously conducted longitudinal studies of adults with chronic LBP^62^^,^^64^ reported that the MIC for the WHODAS 2.0 12-item ranged from −3.22/48 (95% CI −4.79 to −1.64) to −5.99/48 (95% CI −7.20 to −4.79).^52^ Therefore, a MIC value of 6 is proposed for this indicator. It is a conservative measure of improvement in functioning because it was derived using a LBP-specific measure of functioning (ODI).

**Fig. 3:**
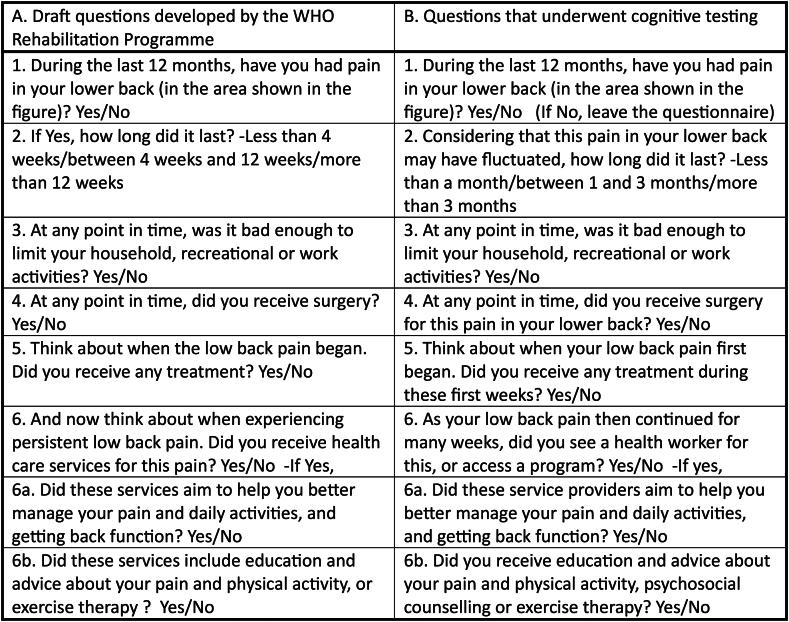
Development of questions.

Fig. 3 presents the draft questions developed by the WHO Rehabilitation Programme and the modifications made following consultation of WHO experts and the external advisory group to develop the list of questions that underwent cognitive testing. Draft question 2 was changed to include potential fluctuations of the pain and to identify the duration of the pain based on months instead of weeks. For question 4, it was decided to ensure that the surgery received was for the pain in the lower back. Question 5 was refined to ask about any treatments during the (sub)acute phase of the LBP and question 6 focussed on continued chronic pain and access to any health worker or, in some countries, a program. Finally, we added psychosocial counselling as key intervention in addition to education and exercise therapy to question 6b.

**Fig. 4:**
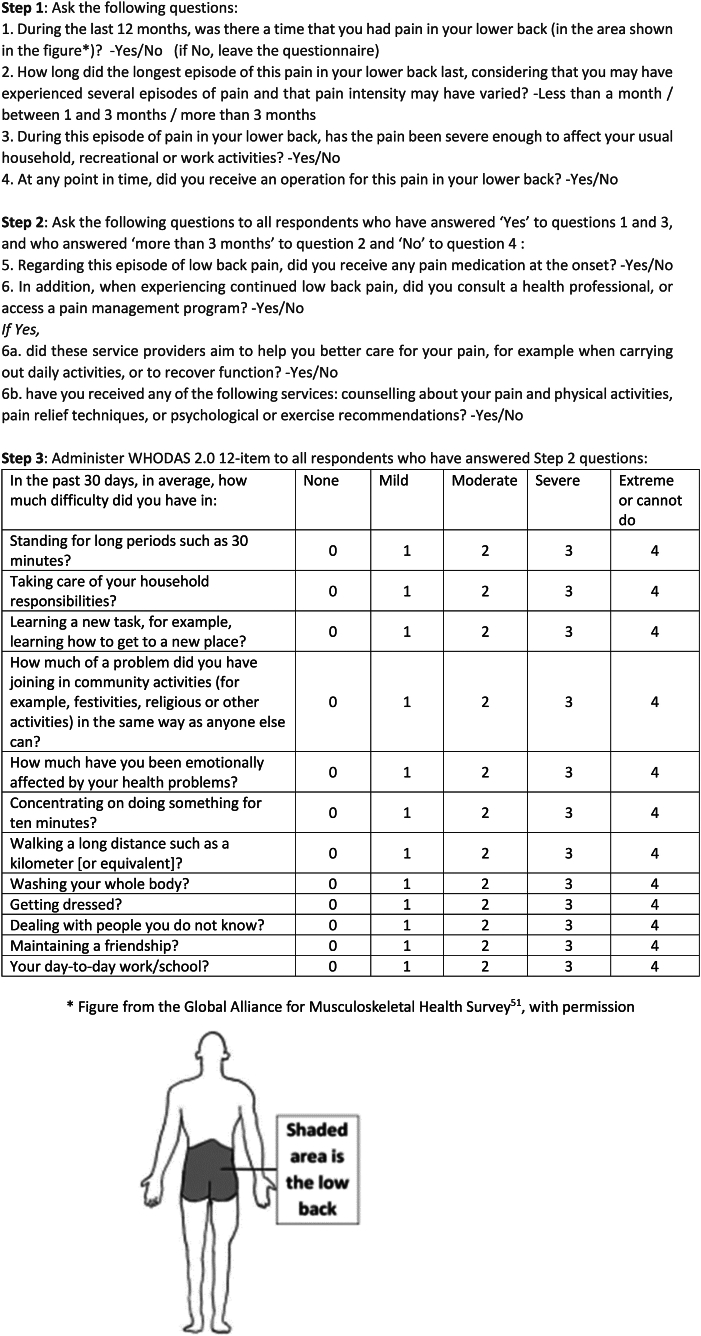
Questions to calculate effective coverage of rehabilitation for chronic primary LBP.

The proposed final list of questions for collecting key data points for the indicator and showing the changes that have been made following cognitive testing is shown at Fig. 4. Main changes after cognitive testing included clarifying question 2 to explain that patients may have experienced recurrent episodes of LBP in the last 12 months but that the aim is to identify the duration of the longest episode. In question 4, we changed receiving surgery to receiving an operation. Question 5 was rephrased to clarify access to treatment at the onset of the pain episode and ‘treatment’ defined as ‘medication’ for better clarity. For question 6, access to a ‘health worker or a program’ was changed to a ‘health professional or pain management program’ to improve the understanding. For question 6a, it was suggested to rephrase ‘manage your pain and daily activities’ to ‘care for your pain, for example when carrying out daily activities’. Finally, for question 6b, we included pain relief techniques as key interventions for rehabilitation of chronic LBP because this would help with capturing utilization of rehabilitation services across countries and regions.

The indicator is calculated as follows: respondents who have answered ‘yes’ to questions 1 and 3, and who answered ‘more than 3 months’ to question 2 and ‘no’ to question 4 define the denominator. Then, the answer to question 5 is not used for calculating the indicator but those responding ‘yes’ to question 6 and ‘yes’ to question 6a and/or 6b are classified as individuals that have received rehabilitation. The average WHODAS 2.0 12-item score of those individuals that have not received rehabilitation is calculated to serve as the baseline functioning score. Individuals that have received rehabilitation and have a WHODAS 2.0 12-item score lower than this baseline score minus 6 are classified as ‘benefited from services’. The numerator is then calculated by counting the number of individuals who benefited from services.

## Discussion

We propose a global indicator to measure effective coverage of rehabilitation at the population level, using chronic primary LBP as the tracer health condition. It is defined as the proportion of adults with chronic primary LBP experiencing limitations in functioning that benefits from rehabilitation. A benefit from rehabilitation is defined by an improvement in functioning based on a Minimal Important Change measured with WHODAS 2.0 12-item. This paper describes the initial steps in the development of the questions to provide the key data points for calculating the indicator through population-based surveys. Next steps of questionnaire development are needed to comply with COnsensus-based Standards for the selection of health Measurement Instruments (COSMIN). Validation studies need to focus on content validity, cross-cultural validity, and measurement error and reliability.

Following further testing and when reliable data become available, rehabilitation coverage could become part of any measurement framework assessing country progress towards UHC. In 2014, WHO and the World Bank published a UHC measurement framework (the official UN measure for the Sustainable Development Goal indicator 3.8.1) which defined service coverage as a spectrum of services provided across the life cycle: promotion, prevention, treatment, rehabilitation, and palliation.^72^ The identification of rehabilitation service coverage bottlenecks, particularly among specific subpopulations or geographic areas, is key to making progress towards UHC.^9^ The WHO UHC measurement framework, and other frameworks that have followed, however, do not include a rehabilitation indicator yet. For now, little data on rehabilitative services across countries and over time has hindered the inclusion of direct or suitable proxies of effective coverage indicators.

The indicator creates an opportunity for rehabilitation because most health data systems in countries are not able to capture all three effective coverage components together with one data source (intervention need, utilization, and quality), especially not at population level.^7^ Although effective coverage indicators are preferred to track progress towards UHC,^7^^,^^73^ most measurement frameworks still rely on other measures, such as crude service coverage indicators, mortality-based measures,^74^ disease prevalence rates and health system core resources densities, to approximate access to quality care. This is in part due to ex post approaches that are used to estimate effective coverage^22^ and their data challenges that sometimes may be managed with triangulation of data sources such as administrative records, health facility records, and community-based surveys.^9^ Administrative health databases, for example, generally offer complete records of intervention use over time but often experience a variety of reporting biases and may not directly link the receipt of interventions to an individual's need for it. In addition, it is important to capture the potential health gains delivered by health systems at the population level.^74^ Therefore, since our set of questions can be integrated into a single household survey, regular survey administrations to measure our effective coverage indicator would overcome this data challenge and bridge the gap as seen in many health programmes.

Measuring effective coverage of rehabilitation is important for understanding whether rehabilitation services are of sufficient availability and quality to produce health gains for the population. Conceptually, effective coverage links potential health gains with health systems inputs and processes. For rehabilitation, this means linking the improvement in functioning of the population with the level of integration of affordable qualitative rehabilitation services across the levels of healthcare. Given its adequate psychometric properties, the proposed indicator uses WHODAS 2.0 to measure an improvement in functioning in a population with chronic primary LBP. For cost-utility analysis, WHODAS 2.0 scores can be used to generate disability weights. A change in the WHODAS 2.0 score for individuals who have received rehabilitation, hence, can be converted in Disability-Adjusted Life Years (DALYs) that have been saved following rehabilitation. Therefore, based on large scale population surveys for this indicator, estimates from individuals can be aggregated and used to quantify gains in population health.^75^ Given the cross-cultural validity of WHODAS 2.0,^76^^,^^77^ this can be done in all countries, including low- and middle-income countries.

With 1 in 3 persons who could benefit from rehabilitation globally,^4^ an important contribution of rehabilitation services will become evident in terms of potential population health to gain once the indicator is included in national effective coverage measurement frameworks. Including the measurement of effective coverage of rehabilitation into a national effective coverage measurement framework is important for understanding whether health services are responsive to the country's health profile and potential health gains. For example, in a recent study, many countries showed lagging performance on effective coverage indicators for non-communicable diseases relative to those for communicable diseases and maternal and child health, despite non-communicable diseases accounting for a greater proportion of potential health gains, suggesting that many health systems are not keeping pace with the rising non-communicable disease burden and associated population health needs.^74^ All health systems ought to maximise potential health gains for their populations and account for local health needs and disease burden, including needs for rehabilitation. Understanding the extent to which health systems are delivering key interventions to those who will benefit from them and the factors that explain gaps in delivery are critical inputs to decision-making at the local, national and global levels.^20^

The use of MIC to define a benefit from rehabilitation at population level needs to be put into perspective. MICs are meant to be used at the individual level with indicators of change measured at two or more time points following rehabilitation.^78^ Because this is not possible in cross-sectional surveys, there is potential for misclassification bias. However, selecting a MIC of 6 to estimate the effectiveness of rehabilitation at population level may reduce the risk of overestimating effective coverage. Moreover, because the WHODAS 2.0 12-item asks about an average level of difficulties with different activities in the past 30 days, selecting the upper MIC value is conservative and accounts for the course of limitations in functioning experienced by people with chronic primary LBP.

In the context of studying the proposed measurement and utilising the indicator for analysis, some elements need to be considered. First, it is important that the surveyed study samples be large enough to provide a precise estimate of the population baseline functioning score. To facilitate the measurement and monitoring of intervention effectiveness with precision, large samples that are recruited on a periodic basis may be required. In addition, an important caveat when comparing health outcomes of individuals with and without exposure to the interventions is the presence of unmeasured confounding as the analyses are observational in nature, and confounding needs to be accounted for.^7^ For example, failure to control for psychosocial prognostic factors or to provide the optimal rehabilitation dosage may lead to biased estimates of effects.

In terms of limitations of this study, some relate to the development of the questions including the absence of consumers in its design and only cognitively tested in two out of six WHO regions. An effort, however, has been made to use as much as possible adaptations from existing survey questions and to confirm face validity through a 2-step process: expert consultation to evaluate and confirm face validity and a cognitive testing process in chronic LBP patients with three different languages (Portuguese, Mandarin, and Cantonese Chinese) to ensure understanding of questions and response options.

Other limitations need to be considered when implementing the proposed method for measuring effectiveness of rehabilitation at population level. These relate to the use of individual level assessments of effectiveness prior to their aggregation for calculating a population estimate. First, an individual may only be classified as a prevalent case that has benefited from services when experiencing no or mild limitations in functioning following rehabilitation. When measured using WHODAS 2.0, a scoping review reported that moderate limitations in functioning are experienced by persons with LBP when seeking and accessing rehabilitation, although getting worse when developing chronic LBP.^67^ When the MIC is subtracted from this baseline score, rehabilitation users would have to experience no or mild limitations in functioning to be classified as ‘benefited from services’. This finding may be in contrast with clinical expertise where individual patients may not experience such a high level of functioning following rehabilitation, yet, benefited from services because they experienced severe limitations in functioning at baseline. When calculating an estimate of effectiveness at population level, this is, however, accepted based on the aggregation of data using a conservative approach for a larger sample in the population. Second, effect size of rehabilitation for chronic primary LBP may be larger in the short- and medium-term than the long-term.^79^^,^^80^ Thus, smaller long-term benefits may be recorded in an individual if rehabilitation was provided 6 months prior to the time point of survey administration. For this reason, some individuals may not be recorded as ‘benefited from services’ while they would have been if surveyed several months earlier. Third, our source population includes people with chronic primary LBP with or without radiating leg pain. Less evidence exists, however, towards the effect of rehabilitation for people with radiating pain. People with chronic primary LBP with radiating leg pain, hence, may benefit less from rehabilitation and to some extent lower the population estimate of effective coverage.

## Contributors

WDG has been technically leading the development of the indicator and questions. WDG, HW, SC, LR, MC, AD, and AC formed the WHO steering committee. PC, JW and SH have systematically reviewed the evidence and constructed MIC data. TC, LB, DK, GW and WDG have been leading on cognitive testing. PC, JW, SH, JH, JH, RM, HA, FR, MN, RL, and J-YL formed the external advisory group. WDG, HW, SC, LR, MC, AD, AC, PC, JW and SH had access to study data and verified the data. WDG wrote the draft manuscript, which was edited and reviewed for important intellectual content by all authors. All authors read and approved the final version of the manuscript.

## Data sharing statement

Not applicable.

## Declaration of interests

JW received a Canadian Chiropractic Research Foundation research grant (paid to institution). JH received a Canadian Institutes of Health Research (CIHR) research grant (paid to institution) and has several partners on CIHR grant applications without personal financial benefits.
